# Biologically Active Extracts from Different Medicinal Plants Tested as Potential Additives against Bee Pathogens

**DOI:** 10.3390/antibiotics10080960

**Published:** 2021-08-10

**Authors:** Claudia Pașca, Ioana Adriana Matei, Zorița Diaconeasa, Ancuța Rotaru, Silvio Erler, Daniel Severus Dezmirean

**Affiliations:** 1 Department of Apiculture and Sericulture, Faculty of Animal Sciences and Biotechnologies, University of Agricultural Sciences and Veterinary Medicine Cluj-Napoca, Calea Mănăștur 3-5, 400372 Cluj-Napoca, Romania; ddezmirean@usamvcluj.ro; 2 Department of Microbiology, Faculty of Veterinary Medicine, University of Agricultural Sciences and Veterinary Medicine Cluj-Napoca, Calea Mănăștur 3-5, 400372 Cluj-Napoca, Romania; ioana.matei@usamvcluj.ro; 3 Department of Chemistry, Biochemistry and Molecular Biology, Faculty of Food Science and Technology, University of Agricultural Sciences and Veterinary Medicine Cluj-Napoca, Calea Mănăștur 3-5, 400372 Cluj-Napoca, Romania; zorita.sconta@usamvcluj.ro; 4 Department of Fundamental Science, Faculty of Animal Science and Biotechnology, University of Animal Sciences and Veterinary Medicine 3-5, 400372 Cluj-Napoca, Romania; ancuta.rotaru@usamvcluj.ro; 5 Julius Kühn Institute (JKI)-Federal Research Centre for Cultivated Plants, Institute for Bee Protection, Messeweg 11-12, 38104 Braunschweig, Germany; silvio.erler@julius-kuehn.de

**Keywords:** *Nosema ceranae*, American foulbrood, chalkbrood, bee diseases, natural antibiotics, alternative treatment

## Abstract

Honey bees (*Apis mellifera*) perform pollination service for many agricultural crops and contribute to the global economy in agriculture and bee products. However, honey bee health is an ongoing concern, as illustrated by persistent local population decline, caused by some severe bee diseases (e.g., nosemosis, AFB, EFB, chalkbrood)**.** Three natural recipes are in development based on the bioactive compounds of different plants extract (*Agastache foeniculum, Artemisia absinthium, Evernia prunastri, Humulus lupulus, Laurus nobilis, Origanum vulgare* and *Vaccinium myrtillus*), characterised by HPLC-PDA. The antimicrobial activity of these recipes was tested in vitro against *Paenibacillus larvae, Paenibacillus alvei, Brevibacillus laterosporus, Enterococcus faecalis, Ascosphaera apis* and in vivo against *Nosema ceranae.* A mix of 20% blueberry, 40% absinthium, 10% oakmoss, 10% oregano, 10% Brewers Gold hops, 5% bay laurel and 5% anise hyssop extract showed the strongest antibacterial and antifungal activity. Combing several highly active plant extracts might be an alternative treatment against bee-disease-associated parasites and pathogens, in particular to replace synthetic antibiotics.

## 1. Introduction

Honey bee diseases are still a serious problem causing local decline in honey bee populations worldwide. Numerous pathogens and parasites are threatening the health and optimal maintenance of honey bee populations, including bacteria (*Paenibacillus larvae* and *Melissococcus plutonius*), fungi (*Ascosphaera apis*), microsporidia (*Nosema apis* and *Nosema ceranae*), trypanosomes (*Crithidia mellificae* and *Lotmaria passim*) and mites (*Varroa* spp. and *Tropilaelaps* sp.) [1,2,3,4,5,6,7,8]. The microbial balance of honey bees, in particular their gut bacterial symbionts—the possible pathogen counterpart, is influenced by a variety of agricultural, colony developmental and environmental factors [9]; such as contaminated water, food or flowers, cleaning activities and trophallaxis, as well as bee faeces deposited in/on the hive or material with infective spores from infected worker honey bees.

*Paenibacillus larvae* is a gram-positive endospore-forming bacterium, the causative agent of American foulbrood (AFB) disease, which infects the brood stages of honey bees (*Apis mellifera* L.). Infected honey bee brood turns black with a spotted appearance and foul smell [1,2,3,4]. Spores of *P. larvae* are resistant to hot, cold, humid and drought conditions, and they may remain infectious for more than 35 years [10,11,12,13]. European foulbrood (EFB) is also a bacterial brood disease caused by the gram-positive bacterium *Melissococcus plutonius,* and associated secondary invaders, such as *Brevibacillus laterosporus, Enterococcus faecalis, Paenibacillus alvei* and *Achromobacter eurydice* have been discussed [2,3]. Chalkbrood is a fungal bee disease caused by *Ascosphaera apis,* which infects young honey bee brood. The disease is initiated at the larval stage when bee larvae are infected with spores carried in pollen or spread by the nurse bees. Larvae mummified by chalkbrood often look like tiny sticks of chalk. Mummies are white, black or grayfish and mottled [8,14].

*Nosema ceranae* is a unicellular fungal parasite that infects the midgut epithelial cells of honey bees [15,16]. The parasite was introduced to European honey bees from its Asian congener (*Apis cerana*) within the last few decades [17,18], and the disease is associated with colony depopulation and collapse in warmer areas of Europe [19]. *N. ceranae* spores germinate in response to proper pH and ionic conditions in the midgut and inject the infective sporoplasm into the host’s cells through extruded polar filament [20,21]. Another parasitic bee disease is varroosis caused by the ectoparasitic mite *Varroa destructor*. There are two distinct phases in the life cycle of *V. destructor* females: A phoretic phase on adult bees and a reproductive phase within the sealed drone and worker brood cells. The mites suck substantial amounts of hemolymph and fat body from both the adult bees and the preimaginal host stages within the sealed brood cells [5,7].

The honey bee (*A. mellifera*) is the most important commercially managed pollinator, with its services to agriculture estimated to exceed $200 billion per year [22,23,24], making bee diseases an important source of economic loss. Beekeepers have some control options for these pathogens, antibiotics such as oxytetracycline, sulfathiazole [25,26,27] and fumagillin [28,29], or biological and synthetic acaricides. The repeated use of synthetic antibiotics to treat bee parasites and pathogens for a long period may have caused multidrug resistance in causative organisms [11], which will require increasing doses of antibiotics, leading to the accumulation of large amounts of antibiotics in beehive products. However, current European legislation does not permit the use of antibiotics for treatment of honey bees [30], because bee products are not allowed to contain drug residues. This prohibition has greatly limited the possibility of disease treatments, but at the same time, stimulated the development of alternative methods against bee pathogens. The replacement of synthetic chemical drugs with natural antimicrobial substances is one promising direction [31]. Beekeepers generally have no desire to contribute to antibiotic resistance, to pollute the environment or to feed synthetic chemicals to their bees [32].

In this context of increasing multidrug resistance and the demand for organic products, the search for alternative drugs based on the pharmacological and phytochemical properties of medicinal additives became a priority in honey bee colony health research. Previous studies have assessed the antimicrobial effect of several natural products based on medicinal plants, for example, laurel leaf extracts against *P. larvae* [12,33] and against bee viruses such as BQCV [34]. Other natural extracts have been reported to inhibit the growth of *A. apis* [25,35]. Moreover, an infusion of *Origanum vulgare* exhibits maximum activity against *B. laterosporus,* essential oil of *O. vulgare* against *Staphylococcus saprophyticus* [36] and essential oil of *Cryptocarya alba* against *N. ceranae* [37]. In several studies, essential oils of mint, melissa, coriander and thyme [38]; other phytochemicals, including caffeine, clove oil, gallic acid, kaempferol and *p*-coumaric acid; and even propolis showed effective antimicrobial activity, expanded honey bee longevity and, to some extent, reduced nosemosis levels [37,39,40,41,42]. Other natural products tested as additives in honey bee feeding, such as BioPatty, which contains a mixture of *Lactobacillus plantarum* Lp39, *Lactobacillus rhamnosus* GR-1 and *Lactobacillus kunkeei* BR-1, could reduce pathogen loads, up-regulate the expression of key immune genes and improve survival during *P. larvae* infection [43,44,45]. Hydroalcoholic laurel extract [12], *Cinnamomum zeylanicum, Syzygium aromaticum*, propolis and *Thymus vulgaris* are active against *M. plutonius* [46]. Medicinal plants (*Eugenia caryophyllus*, *Piper betel*, *Illicium verum*, *Cinnamomum cassia* and *Acorus calamus*) at various concentrations (0.25–10%) were assayed against *A. apis* [14], as well as propolis extracts [47] and another plant extract [25]. The most popular alternative treatments against nosemosis in Europe are: Protofil, a mixture of macerated plants; Nozevit, a natural extract of oak bark; Hive alive(contains seaweed extracts); *Artemisia absinthium* extract; propolis extract; laurel; oregano; rosemary; cinnamon; and eucalypt essential oils, which have showed adequate antimicrobial activity against this pathogen [26,33,34,35,36,37,42,48,49,50,51].

When we began this study, we started with the idea that on the market there is Protofil, a product obtained from macerated plant extracts; a number of 10 plants were used, with alcohol at 80%. In our study we selected the most efficiently medicinal plants and we separated the extract. We took for the extracts only the part of plants with biologically active compounds. We made three recipes based on these extracts but at different concentrations. We chose the concentration of plant extracts for the recipes after the biologically compounds identified in our experiment and also based on the fact that many plant extracts include secondary compounds, which are toxic to insects, and for this reason we have different concentrations.

The development of new recipes and testing their effectivity against bee parasites and pathogens is an alternative way to overcome resistance to classical treatments against mites, bacterial infections, viruses and fungal bee disease. Essential oils from medicinal plants have proven effectivity against bee diseases; however, only a few products are available on the market. Here, we estimate the biologically active compounds of several medicinal plant extracts and tested new recipes, which might be used as additives in honey bee feeding in order to control bee pathogens.

## 2. Results

### 2.1. Total Phenolic, Flavonoid Content and Phenolic Compounds

**Figure 1:**
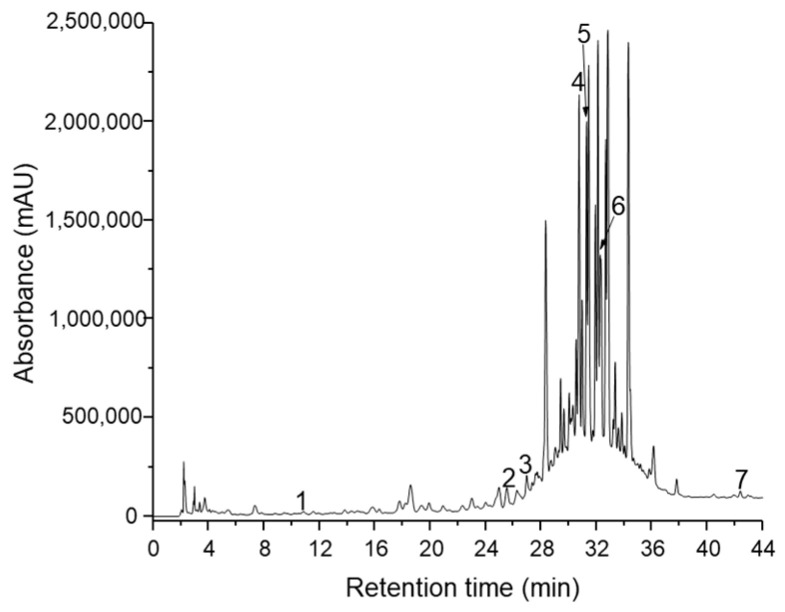
Chromatogram of identified polyphenolic compounds detected in extracts of *Laurus nobilis* (1-*p*-OH-benzoic acid; 2-syringic acid; 3-epicatechina; 4-rutin; 5-isoquercitrin; 6-quercetin; 7-kaempferol).

**Figure 2:**
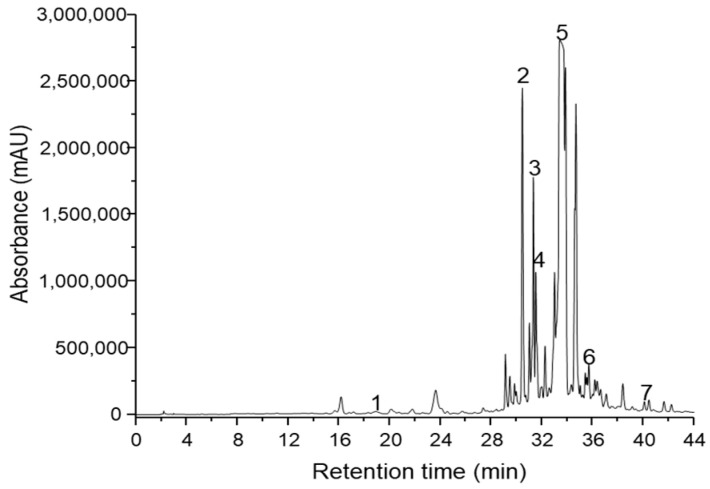
Chromatogram of identified polyphenolic compounds detected in extracts of *Origanum vulgare* *(*1-p-OH-benzoic acid; 2-vitexin 2-O-rhamnoside; 3-sinapic acid; 4-isoquercitrin; 5-rosmarinic acid; 6-resveratrol; 7-apigenin).

A sensitive, accurate and specific method coupling HPLC with photodiode array detection (PDA) and spectrometry was developed for the analysis of phenolic and flavonoid compounds in extracts of *Agastache foeniculum, Artemisia absinthium, Evernia prunastri, Humulus lupulus, Laurus nobilis, Origanum vulgare, Vaccinium myrtillus* and sunflower honey. Phenolic and flavonoid compounds were identified on the basis of their retention time, absorbance spectra and a chromatogram with authentic standards. Figure 1 and Figure 2 show the HPLC chromatogram for the extract of *Laurus nobilis* and *Origanum vulgare.* The total phenolic and flavonoid content were identified using the Folin–Ciocalteu method and aluminium chloride colorimetric method.

Mobile phases have been formed by water at pH 2.5 adjusted with *ortho*-phosphoric acid (solvent A) and acetonitrile (solvent B). Elution was carried out using a flow rate of 1 mL/min at 24 °C. The chromatogram was made at 254 nm and the compounds were identified at characteristic wavelengths for each compound.

Different quantities of total phenolic content (1.831–44.672 mg GAE/g DW) and flavonoids (2.215–30.520 mg QE/g DW) were obtained for each specific plant extract (Table 1). The highest concentration of phenolic content was identified for *O. vulgare*, and for the flavonoid content for *A. absinthium.*

Among the 25 identified phenolic compounds (Table 2), some of them are already known for their importance on bee health, such as: chlorogenic acid [52] and resveratrol [53,54]. Isoquercitrin and *p*-OH-benzoic acid were identified in different amounts as a major compound in all plant extracts. Dominating quantities of chlorogenic acid (312.18 µg/g DW) were measured in *V. myrtillus* extract, rutin (523.25 µg/g DW) and epicatechin (501.29 µg/g DW) in *H. lupulus* extract, rosmarinic acid (633.85 µg/g DW) and vitexin 2-O-ramnoside (470.45 µg/g DW) in *O. vulgare* extract, rutin (869.21 µg/g DW) and isoquercitrin (577.15 µg/g DW) in *L. nobilis* extract, and quercetin (389.73 µg/g DW) in *A. absinthium* extract.

The highest concentration (µg/g DW) of total phenolic acids and total flavonoids was identified in the *L. nobilis* extract (Figure 1) and the *O. vulgare* extract (Figure 2).

Mobile phases have been formed by water at pH 2.5 adjusted with *ortho*-phosphoric acid (solvent A) and acetonitrile (solvent B). Elution was carried out using a flow rate of 1 mL/min at 24 °C. The chromatogram was made at 254 nm, and the compounds were identified at characteristic wavelengths for each compound.

### 2.2. Antioxidant and Antimicrobial Activity

The antioxidant activity of the medicinal plant extracts and sunflower honey were performed by DPPH-radical scavenging activity [41,55] and the cupric reducing antioxidant capacity (CUPRAC) method [56]. All plant extracts were characterised for their antioxidant capacity (Table 3) as well as their in vitro antimicrobial activity against bee-disease associated-bacteria and fungi (Table 4). The highest percentage of the inhibition of the DPPH radical (97.50%) was detected for the extract of *A. absinthium*, which also exhibited the strongest antibacterial activity on *P. larvae* and *E. faecalis* (Table 4). For the total antioxidant power, CUPRAC value, the order is slightly changed. The *O. vulgare* extract showed the highest concentration (165.59 µmol TE/g DW), followed by the *L. nobilis* extract (39.16 µmol TE/g DW). 

The antimicrobial activity of the medicinal plant extracts was evaluated on selective medium plates by the agar well diffusion method [41].

The highest antibacterial activity against *P. larvae* was observed for extracts of *A. foeniculum* and *A. absinthium* and against *P. alvei* and *B. laterosporus* for *H. lupulus* extract (Table 4). *A. absinthium* and *L. nobilis* extracts showed strongest antifungal activity against *A. apis*.

### 2.3. Medicinal Additive Recipes

The efficacy of chemical constituents of plants depends on the mode of preparation and conservation of its extracts. Many plant extracts include secondary compounds that are toxic to insects. Honey bees usually avoid contact with nectar that contains such secondary compounds, but here, diet selection was not allowed [57]. Based on the results of their biological activity (antioxidant activity, phenolic acids and flavonoids), compound composition of the plant extracts and results of previous studies [55], three medicinal recipes were developed using different concentrations of the mentioned plant extracts (Table S1).

All recipes (R1, R2, R3) were tested in terms of their antioxidant and antimicrobial potential before running the in vivo experimental study using *N. ceranae*-infected bees. The highest concentration of the antioxidant activity (CUPRAC value) was estimated for recipe 2 (119.4 µM TE/100 g), followed by recipe 3 (112.5 µM TE/100 g) and recipe 1 (94.4 µM TE/100 g). Differences between different natural recipes may be explained by the different concentrations of their ingredients (Table 2, Table S1), in particular phenolic, polyphenolic and flavonoid total contents: R2 (895.128 µg/g DW) > R3 (879.410 µg/g DW) > R1 (838.783 µg/g DW).

Regarding the antimicrobial activity, recipe 2 showed the strongest activity, followed by recipe 3 and recipe 1. Recipe 2 presented means of inhibition areas comparable with the positive control in most cases. Among the tested bacterial strains, the most sensitive were *P. alvei* and *B. laterosporus* (Table 4).

### 2.4. Effectivity against N. ceranae

Bees that died during the experiment or survived until the end of the experiment were collected for each cage on day 7, day 14 and day 21 post-infection. The spore levels of surviving bees at the end were as follows; recipe 2: 2257 spores/bee (C4, C7), recipe 3: 2587 spores/bee (C2, C3), sunflower honey: 2895 spores/bee (C1), Protofil: 2979 spores/bee (C5) and recipe 1: 4830 spores/bee (C8), while the untreated bees showed a spore level of 124,400 spores/bee (C6) (Table S3). All treated bees that died during the experiment showed much lower spore loads than the non-treated control (day 14 and 21), with decreasing spore numbers from day 7 to day 21. Bees that survived until day 21 were shown to have the lowest spore loads compared to the non-treated control and bees that died earlier (Table S3).

The same results were observed for the presence of *Nosema* spp. and *N. ceranae*-specific PCR. All positive samples for *Nosema* spp. (16S rRNA gene fragment) were also positive for the *N. ceranae* 16S rRNA gene fragment, confirming an infection with *N. ceranae.* Bees collected prior to the experimental infection were PCR negative (*n* = 0/10), and all infected and untreated bees (positive control) were positive (*n* = 10/10). Overall, 36.84% (*n* = 42/114) of the bees yielded a negative PCR-result after infection and treatment. The highest rate of negative bees was obtained after treatment with recipe 2 plant extract, 56.25% (*n* = 18/32) (Figure S1), followed by Protofil (*n* = 6/13, 46.15%), recipe 3 (*n* = 13/34, 38.24%), recipe 1 (*n* = 2/11, 18.18%) and sunflower honey (*n* = 2/12, 16.67%) (Figures S1 and S2). Finally, recipe 2, 3 and Protofil were more active against *N. ceranae* than recipe 1 and sunflower honey.

## 3. Discussion

The present study attempts to be part of the replacement of synthetic antibiotics, which are used in treating bee diseases (nosemosis, AFB, EFB, chalkbrood), with proposing medicinal additive recipes composed of plants extracts or with biologically active compounds of different plant extracts. The effectiveness of the three recipes obtained in the present study was analysed using an in vitro and in vivo approach. However, more colony replicate cage studies, larvae assays and finally, colony assays are needed to finally confirm their activity under field-realistic conditions. In particular, the results for *N. ceranae*-infected bees have to be seen as preliminary due to reduced replicate and sample sizes.

All the recipes showed strong antimicrobial activity, and each plant extract was characterised for its individual polyphenolic composition. The mechanism by which plant extracts of blueberries, absinthium, oakmoss, oregano, Brewers Gold hops, bay laurel and anise hyssop employed their antimicrobial activity might be attributed to their bioactive components (phenolic acids and flavonoids) as their active biomolecules. The obtained results have shown an inhibition of bacterial (AFB and EFB) and fungal (nosemosis and chalkbrood) growth for all tested plant extracts, with best results for *A. absinthium,* *H. lupulus*, *O. vulgare* and *V.* *myrtillus.* To our knowledge there are not many studies on the effectivity of alcoholic extracts of blueberries, absinthium, oakmoss, oregano, Brewers Gold hops, bay laurel and anise hyssop on bee pathogens [33,34,36]. Nevertheless, the results of the current study are in line with results of many other plants extracts, which are used against bee parasites and pathogens (nosemosis, AFB, EFB, chalkbrood), such as: *Aristotelia chilensis, Gevuina avellana, Ugni molinae* and propolis methanolic extracts [40] or *Buddleja thyrsoides* [58] and *Scutia buxifolia* [59]; furthermore, some studies use *Humulus lupulus* and *Myrtus communis* [60], *Origanum vulgare* and *Rosmarinus officinalis* [61] or *Laurus nobilis* [12,33,34]. However, others showed that bay laurel oil and phenolic extracts of berries had very low or no activity on Gram-positive bacteria [11]. 

Scientists and beekeepers alike are keen on finding new natural recipes of products that improve bee health. One way to overcome the huge variety of natural products and active ingredients is to combine naturally produced compounds [32]. Therefore, a major objective of this study was to create new recipes based on biologically active compounds from different medicinal plants that can be used as additive products in honey bee feeding for the control of bee diseases. The most efficient recipe on the control of bacterial and fungal pathogens was recipe 2. Differences between the recipes were due to the concentrations in bioactive compounds and ingredients. In Europe [42,49,50], beekeepers use Protofil for the control of nosemosis. Protofil is a maceration of several plants (chamomile, St. John’s wort, marigold, mouse tail, thyme, basil, mint, sea buckthorn, rosehip, plantain, wormwood, acacia flowers, linden) in alcohol at 70 °C. The medicinal additive recipes (R1, R2, R3) were tested together with Protofil and sunflower honey on honey bees artificially infected with *N. ceranae*. However, no treatment resulted in 100% negative PCR results. Using PCR for evaluating treatment efficiency should be considered with caution as *N. ceranae* DNA will be amplified regardless of the viability of spores. Furthermore, using cage assays, infected bees do not have the opportunity to get ride of the infective spores (by defaecation) even if they are potentially inactivated by the bioactive medicinal additive.

Beekeepers have some opportunities to control bacterial infections (AFB, EFB) with antibiotics such as oxytetracycline or sulfathiazole, or fumagillin against *Nosema* sp., but no registered product exists to control chalkbrood. Currently, there is no product that can be used for treating honey bees against several bee diseases in one application. Recipes tested and developed in this study might be applied to control nosemosis, AFB, EFB and chalkbrood. They are made based on the individual extraction of each plant. The selection of plants should be based on their antioxidant and antimicrobial potential, as well as having no effect harming honey bees.

## 4. Materials and Methods

### 4.1. Plant Material and Ethanolic Extracts

The plant material was harvested from the research field of the University of Agricultural Sciences and Veterinary Medicine Cluj-Napoca in 2018. All plant material was dried at room temperature. Bioactive compounds from different parts of the plants (*Agastache foeniculum, Artemisia absinthium, Evernia prunastri, Humulus lupulus, Laurus nobilis, Origanum vulgare* and *Vaccinium myrtillus*, Table S2) were extracted using a maceration technique with 80% ethanol (*v*/*v*), continuous stirring and protection from light for 96 h to a final concentration of 1% extract [62].

### 4.2. Total Phenolic, Flavonoid Content and Polyphenolic Composition

The total polyphenolic content was estimated using the Folin–Ciocalteu method (S.I.1.) and was expressed as gallic acid equivalents (GAE) in mg/g dry weight of medicinal plants [55,63,64]. The total flavonoid content (expressed as quercetin equivalent (QE) in mg/g dry weight of medicinal plants) was determined by the aluminium chloride colorimetric method (S.I.2.) as described previously [55,63]. All samples were analysed in triplicate.

Individual polyphenolic characterisation of each plant extract was conducted on a liquid chromatograph Shimadzu 2010 EV (Kyoto, Japan) coupled with an SPD-M20A Photodiode Array Detector, LC-10ADSP binary pumps, a CTO-10AVP column oven and an SIL-10AF autosampler [65]. Compound separation was achieved on a Teknokroma Mediterranean Sea C18 column (15 × 0.46, i.d. 5 µm). Mobile phases have been formed by water at pH 2.5 adjusted with *ortho*-phosphoric acid (solvent A) and acetonitrile (solvent B) using a binary gradient unit LC-10AD VP. Elution was carried out using a flow rate of 1 mL/min at 24 °C and an injection volume of 10 µL. Spectral data for all peaks were accumulated in the range of 220–400 nm [66]. Concentration of each individual compound was calculated based on an external standard method, using calibration curves for each compound, after comparing retention time and UV spectra with reference standards. All chemicals and solvents used were of analytical grade.

### 4.3. Antioxidant Activity

DPPH (2,2-diphenyl-1-picrylhydrazyl) free radical scavenging activity of the medicinal plant extracts was evaluated spectrophotometrically by a method described by Dulf et al. (2018) [64] (S.I.3.). The results were expressed as µmol Trolox equivalents per 100-g sample (µM Trolox/100 g dry weight). The percentage of radical scavenging activity was converted to Trolox equivalents using a calibration curve (y = 0.0009x − 0.0051, R^2^ = 0.9974). The percent inhibition of the radical scavenging activity was calculated by the following formula:

Percentage inhibition (%) = [(A_blank_ − A_sample_)/A_blank_] × 100, where A_blank_ is the absorbance of the control reaction (DPPH alone), and A_sample_ is the absorbance of the DPPH solution in the presence of the test compound [48].

The modified cupric reducing antioxidant capacity (CUPRAC) method was applied to evaluate the antioxidant potential [63] (S.I.4.) of the medicinal plant extracts. The samples were analysed at 450 nm, and measurements were expressed as µmol Trolox equivalents per gram of plant material (μmol TE/g dry weight). All samples were analysed in triplicate.

### 4.4. Antimicrobial Activity

Four bacterial strains (*Paenibacillus larvae* ERIC I genotype–RO-I.D.S.A.-14502/17*, Paenibacillus alvei–*RO-I.D.S.A.-114/09, *Brevibacillus laterosporus–*RO-I.D.S.A.-99-186-22/08*, Enterococcus faecalis–*RO-I.D.S.A.-14490/13) and one fungal strain (*Ascosphaera apis*–RO-I.D.S.A-11885/17) from the Institute of Diagnosis and Animal Health (I.D.S.A) of Bucharest, Romania, were used. The antimicrobial activity of medicinal plant extracts and the three recipes was tested on selective medium plates by the agar well diffusion method [42]. Vegetative cells of bacterial strains and *A. apis* were suspended in 10 mL distilled sterile water (1.5 × 10^8^ CFU (colonies forming units)/mL), confirmed spectrophotometrically at 600 nm). Every petri dish was floated with the bacterial suspension and dried. Nine micro-compartments (discs of filter paper) with plant extracts or mixed products (50 μL each) were distributed on each plate. Agar plates were incubated at 37 °C for 24 h for the bacterial strains and at 25 °C for 48 h for *A. apis*. The results were expressed in mm zone of inhibition. The concentration of the negative control (ethanol) was similar to the concentration used in the plant extracts, for the positive control (oxytetracycline) the concentration was 30 µg per disc.

### 4.5. Nosema Infection Assay

All experiments were carried out in April to May 2019. To minimise any potential colony-level effects, a single sealed worker brood frame was obtained from one healthy, *Nosema*-free (confirmed by PCR) and *Varroa*-free (confirmed by macroscopic examination) colony of *Apis mellifera* located in the experimental apiary (Department of Apiculture and Sericulture, University of Agricultural Sciences and Veterinary Medicine Cluj-Napoca). The frame was kept in an incubator at 34 ± 2 °C until workers emerged.

*Nosema* spores were obtained from a highly infected colony and the bee samples were analysed by the Institute of Diagnosis and Animal Health (I.D.S.A) of Bucharest using a method described by Fries et al. (2013) [67]. Viable *Nosema* spores, isolated from the infected colony and provided by I.D.S.A. Bucharest, were used for the infection of honey bees in this study.

Freshly emerged bees were starved for 3 h and 40 bees per group were fed with 10 µL of 50% sucrose solution (*w*/*v*) containing 10^5^ *Nosema* spores. After infection, the bees were kept in cages for another 7 days to ensure that the spores reached the midgut [19,68,69,70]. Then, 7 days post-infection, natural products based on plant extracts were used as therapeutic additives in honey bee feeding until the end of the experiment. Extracts were supplied in sugar syrup so as to force bees to ingest them in each feeding. Five cages (C2, C3, C4, C7, C8) equipped with 40 bees were assigned to different treatments with medicinal additives at 5% (C2 and C3 with recipe 3; C4 and C7 with recipe 2; C8 with recipe 1); one cage (C1) was assigned to sunflower honey that was shown to be active against *N. ceranae* and honey bee bacterial pathogens [71,72]; two cages were assigned to a commercial product (Protofil–C5) and the untreated control (infested bees and untreated–C6). The negative control was represented by a lot of honey bees without nosemosis, from the original colony of the brood frame. Dead bees were removed and counted on days 7, 14 and 21. Ventriculi of dead bees were checked separately for the presence of *Nosema* spores. Bee samples of bees that survived were collected from each cage on day 21 post-infection.

### 4.6. Determination of Spore Levels

To determine *N. ceranae* infection levels for each cage, dead and survived worker bees were collected post-infection: on day 7 (C1: 5 dead bees, C2: 4 dead bees, C3: 5 dead bees, C4: 2 dead bees, C5: 2 dead bees, C6: 5 dead bees, C7: 1 dead bee, C8: 4 dead bees), on day 14 post-infection (C1: 9 dead bees, C2: 4 dead bees, C3: 2 dead bees, C4: 2 dead bees; C5: 2 dead bees, C6: 12 dead bees, C7: 2 dead bees, C8: 3 dead bees), and on day 21 post-infection (C1: 8 dead bees, 6 survived; C2: 5 dead bees, 12 survived; C3: 6 dead bees, 8 survived; C4: 2 dead bees, 17 survived; C5: 3 dead bees, 20 survived; C6: 3 dead bees, 10 survived bees; C7: 2 dead bees, 20 survived; C8: 7 dead bees, 15 survived). Each worker’s abdomen was homogenised with a pestle, diluted in 1 mL sterile water per bee, filtered through a 10 µm pore size filter paper and finally centrifuged for 20 min at 3900× *g*. The spore pellet was resuspended in 2 mL sterile water, pooled per cage and the number of spores was determined using a Neubauer counting chamber [71].

### 4.7. DNA Extraction and Species Verification

Ten to twenty surviving bees were collected from each cage on day 21 post-infection and stored in 96% ethanol. Each bee was washed in PBS and dried on sterile filter paper before extraction. The bees were cut into two symmetrically halves using sterile single use blades. Genomic DNA was extracted from each bee (both halves) using a commercial kit (ISOLATE II Genomic DNA Kit, Bioline, UK), following the manufacturer’s instructions. In order to assess cross-contamination between extracts, negative controls consisting of reaction mixes without a bee sample were used in each extraction procedure. DNA quantity and purity were determined on a Nanodrop ND-1000 spectrophotometer (NanoDrop Technologies, Inc., Wilmington, DE, USA), using a representative number of randomly selected samples. DNA was stored at −20 °C until further processing.

Samples were assessed for the presence of *Nosema* spp. and in particular *Nosema ceranae,* but not for *N. apis*, as this *Nosema* species has not been detected in the last decades in samples of different counties of Romania [73,74]. Initial PCR for *Nosema* spp. detection was performed using a genus-specific primer set amplifying a 252 bp fragment of the 16S rRNA gene (NOSFOR: TGCCGACGATGTGATATGAG/ NOSREV: CACAGCATCCATTGAAAA CG) [17]. *N. ceranae* DNA detection was realised using a species specific primer set amplifying a 250 bp fragment of the 16S rRNA gene (*N. ceranae* F: CGGATAAAAGAGTCCGTTACC, *N. ceranae* R: TGAGCAGGGTTCTAGGGAT) [75]. Each 25 µL reaction mixture contained 12.5 µL Green PCR Master Mix (Rovalab GmBH), 6.5 µL PCR grade water, 1 µL of each primer (10 µM) and 4 µL isolated DNA. Following optimisation, the amplification profile for *Nosema* spp. detection started with 3 min of initial denaturation at 95 °C, followed by 40 cycles of denaturation at 95 °C for 30 s, annealing at 55 °C for 45 s and extension at 72 °C for 1 min, followed by final extension at 72 °C for 5 min. The amplification profile for *N. ceranae* detection followed the same protocol, except annealing at 45 °C for 45 s. In each PCR assay, positive and negative controls were included in order to assess the specificity of the reaction and potential presence of cross-contamination. Positive controls consisted of DNA extracted from a naturally infected bee, previously confirmed to be *N. ceranae* by sequencing. The negative control was a reaction mix without DNA. PCRs were run using a T100 Thermal Cycler (Bio-Rad *Antibodies Distributor* SC Dialabs Street Albac no 1, Sector 1, Bucharest, Romania). PCR products were visualised by agarose gel electrophoresis (1.5%) stained with SYBR Safe DNA gel stain (Invitrogen).

### 4.8. Statistical Analysis

Significant differences between plant extracts and sunflower honey were analysed with one-way ANOVA post hoc tests, and pairwise multiple comparisons were conducted using Tukey’s test. Zone of inhibition values were shown not to be normally distributed (Shapiro–Wilk test) and did not match criteria for homoscedasticity (Levene test). Results were analysed in STATISTICA 12.0 (StatSoft, Tulsa, OK, USA) using Kruskal–Wallis ANOVA and Bonferroni adjusted Dunn’s post hoc tests. Phenolic and flavonoid compounds and antioxidant activity were analysed using the SPSS programme (version 22.0, Chicago, IL, USA).

## 5. Conclusions

This study showed that biologically active compounds from different medicinal plants might be useful in the control of several bacteria associated with bee diseases, as well as the fungus *A. apis* and infections with *N. ceranae.* Each phenolic compound of each plant extract has its own therapeutic properties for the honey bee colony, and by mixing these compounds, a natural antibiotic extract will be obtained. This natural antibiotic mixture added in artificial honey bee feeding, as food additive, might replace synthetic antibiotics and eliminate the risk of drug residues in bee products. Prophylactic and therapeutic treatments against bee disease will not only reduce disease-associated colony losses but also enhance pollination and ecosystem service of honey bees using healthy colonies.

## 6. Patents

Patent application no. A00994/2018 with title: Phytotherapeutic tincture ”BIONOSEM”.

## Figures and Tables

**Table 1:** Total phenolic and flavonoid content of the different plant extracts and sunflower honey. Results are given as mean values of three independent replicates ± standard deviation.

Sample	Total Phenolic Content mg GAE/g DW	Total Flavonoid Content mg QE/g DW
*Agastache foeniculum*	2.882 ± 0.005 ^c,d^	2.215 ± 0.001 ^d^
*Artemisia absinthium*	1.831 ± 0.03 ^d^	30.520 ± 0.002 ^a^
*Evernia prunastri*	4.574 ± 0.011 ^c^	2.560 ± 0.007 ^d^
*Humulus lupulus*	5.344 ± 0.003 ^c^	2.511 ± 0.001 ^d^
*Laurus nobilis*	14.856 ± 0.003 ^b^	5.685 ± 0.09 ^c^
*Origanum vulgare*	44.672 ± 0.006 ^a^	20.835 ± 0.02 ^b^
*Vaccinium myrtillus*	13.241 ± 0.007 ^b^	5.710 ± 0.005 ^c^
Sunflower honey	3.570 ± 0.05 ^c,d^	1.890 ± 0.004 ^d^

Note: Different letters within a column denote significant differences (*p* < 0.05). For the total phenolic content: ^a^ *Origanum vulgare* and *Artemisia absinthium* different from the others’ samples (*p* < 0.05) and ^b^ *Laurus nobilis, Vaccinium myrtillus* and *Origanum vulgare* different from the others’ samples (*p* < 0.05), ^c^ *Agastache foeniculum*, *Evernia prunastri*, *Humulus lupulus* and sunflower honey different from the others’ samples (*p* < 0.05); ^d^ *Agastache foeniculum, Artemisia absinthium* and Sunflower honey different from the others’ samples (*p* < 0.05); (Results were analysed using SPSS programme); GAE: gallic acid equivalents, QE: quercetin equivalents, DW: dry weight.

**Table 2:** Characteristic phenolic acids and flavonoids of *Agastache foeniculum, Artemisia absinthium, Evernia prunastri, Humulus lupulus, Laurus nobilis, Origanum vulgare, Vaccinium myrtillus* extracts and sunflower honey (expressed as µg/g dry weight).

Identified Compound	*Agastache foeniculum*	*Artemisia*	*Evernia prunastri*	*Humulus lupulus*	*Laurus nobilis*	*Origanum vulgare*	*Vaccinium myrtillus*	Sunflower Honey
Chlorogenic acid	49.88 ± 2.37 ^b^*	n.d.**	1.44 ± 0.07 ^c^*	n.d.**	n.d.**	n.d.**	312.18 ± 15.24 ^a^*	2.95 ± 0.15 ^c^*
Syringic acid	n.d.**	n.d.**	n.d.**	n.d.**	120.68 ± 5.80 ^a^*	n.d.**	76.68 ± 3.88 ^b*^	n.d.**
Ferulic acid	n.d.**	n.d.**	n.d.**	n.d.**	n.d.**	n.d.**	118.50 ± 5.78 ^a^*	0.80 ± 0.04 ^b^*
Isoquercitrin	283.22 ± 14.37 ^c^*	58.53 ± 2.98 ^e^*	n.d.**	298.87 ± 15.47 ^c^*	577.15 ± 29.53 ^a^*	347.90 ± 16.83 ^b^*	149.59 ± 7.20 ^d^*	n.d.**
Quercetin	117.69 ± 6.603 ^c^*	389.73 ± 19.48 ^a^*	n.d.**	n.d.**	419.32 ± 21.19 ^a^*	n.d.**	159.10 ± 8.03 ^b^*	5.99 ± 0.29 ^d^*
Miricetin	n.d.**	n.d.**	n.d.**	n.d.**	n.d.**	n.d.**	10.21 ± 0.51 ^a^*	1.03 ± 0.05 ^b^*
Naringenin	n.d.**	n.d.**	n.d.**	n.d.**	n.d.**	n.d.**	0.08 ± 0.00 ^a^*	n.d.**
Kaempferol	n.d.**	n.d.**	n.d.**	n.d.**	7.46 ± 0.38 ^b^*	n.d.**	0.28 ± 0.01 ^c^*	17.80 ± 0.90 ^a^*
Vanilic acid	n.d.**	n.d.**	1.91 ± 0.09 ^a*^	n.d.**	n.d.**	n.d.**	n.d.**	n.d.**
Vanillin	40.96 ± 1.94 ^a^*	n.d.**	6.41 ± 0.01 ^b^*	n.d.**	n.d.**	n.d.**	n.d.**	n.d.**
Rosmarinic acid	n.d.**	n.d.**	9.45 ± 0.45 ^b^*	n.d.**	n.d.**	633.85 ± 32.99 ^a^*	n.d.**	n.d.**
Crisin	n.d.**	n.d.**	70.94 ± 3.6 ^a^*	n.d.**	n.d.**	n.d.**	n.d.**	n.d.**
o-Cumaric acid	n.d.**	n.d.**	4.48 ± 0.22 ^a^*	n.d.**	n.d.**	n.d.**	n.d.**	1.46 ± 0.07 ^b^*
Acacetin	52.83 ± 2.61 ^a^*	n.d.**	7.55 ± 0.36 ^b^*	n.d.**	n.d.**	n.d.**	n.d.**	n.d.**
Gallic acid	38.86 ± 1.85 ^a^*	n.d.**	n.d.**	n.d.**	n.d.**	n.d.**	n.d.**	0.63 ± 0.03 ^b^*
Caffeic acid	20.34 ± 1.00 ^a^*	n.d.**	n.d.**	n.d.**	n.d.**	n.d.**	n.d.**	2.71 ± 0.13 ^b^*
*p*-OH Cinnamic acid	9.01 ± 0.45 ^a^*	n.d.**	n.d.**	n.d.**	n.d.**	n.d.**	n.d.**	n.d.**
Apigenin	17.38 ± 0.88 ^b^* n.d.**	n.d.**	n.d.**	n.d.**	n.d.**	93.55 ± 4.72 ^a^*	n.d.**	0.93 ± 0.04 ^c^*
Rutin	n.d.**	115.71 ± 5.7 ^c^*	n.d.**	523.25 ± 26.89 ^b*^	869.21 ± 30.22 ^a^*	n.d.**	n.d.**	1.24 ± 0.06 ^d^*
Epicatechina	n.d.**	n.d.**	n.d.**	501.29 ± 24.40 ^a^*	142.82 ± 7.16 ^b^* n.d.**	n.d.**	n.d.**	n.d.**
Vitexin 2-o-ramnoside	n.d.**	n.d.**	n.d.**	n.d.**	n.d.**	470.45 ± 23.25 ^a^*	n.d.**	n.d.**
Sinapic acid	n.d.**	n.d.**	n.d.**	n.d.**	n.d.**	446.86 ± 21.96 ^a*^	n.d.**	n.d.**
Resveratrol	128.59 ± 6.72 ^c^	n.d.**	n.d.**	n.d.**	139.2 ± 6.90 ^c^	75.19 ± 3.63 ^a*^	n.d.**	n.d.**
Total phenolic acids	512.08 ± 25.18 ^d^	28.77 ± 1.39 ^d^	33.94 ± 1.64 ^d^	95.48 ± 4.70 ^c^	2015.96 ± 97.01 ^a^	1095.03 ± 52.96 ^a^	512.02 ± 24.59 ^b^	2.71 ± 0.14 ^d^
Total flavonoids		563.97 ± 29.08 ^d^	84.90 ± 4.26 ^f^	1323.41 ± 68.86 ^b^		987.09 ± 48.27 ^c^	319.26 ± 16.50 ^e^	26.99 ± 1.37 ^f^

* The values are mean of three samples (*n* = 3), analysed individually in triplicates. Different letters within a row denote significant differences (*p* < 0.05); for example, 3,4 dihydroxybenzoic identified in ^a^ *Humulus lupulus* extract, ^b^ *Artemisia absinthium* extract, ^c^ *Agastache foeniculum* and ^d^ *Evernia prunastri* extract is different from the others extracts or sunflower honey (*p* < 0.05); *p*-OH-benzoic acid identified in ^a^ *Humulus lupulus* extract, ^b^ *Laurus nobilis* extract, ^c^ *Origanum vulgare* extract, ^d^ *Artemisia absinthium* and *Evernia prunastri* extract is different from the others extracts or sunflower honey (*p* < 0.05); Chlorogenic acid identified in ^a^ *Vaccinium myrtillus* extract, ^*b*^ *Agastache foeniculum* extract, ^*c*^ *Evernia prunastri* extract and sunflower honey is different from the others extracts (*p* < 0.05); Syringic acid identified in ^a^ *Laurus nobilis* and ^b^ *Vaccinium myrtillus* extract is different from the others extracts or sunflower honey (*p* < 0.05); Ferulic acid identified in ^a^ *Vaccinium myrtillus* extract and sunflower honey is different from the others extracts (*p* < 0.05); Isoquercitrin identified in ^a^ *Laurus nobilis* extract, ^b^ *Origanum vulgare* extract, ^c^ *Agastache foeniculum* and *Humulus lupulus* extract, ^d^ *Vaccinium myrtillus* extract is different from the others extracts or sunflower honey (*p* < 0.05); Quercetin identified in ^a^ *Artemisia absinthium* and *Laurus nobilis* extract, *^b^ Vaccinium myrtillus* extract, ^c^ *Agastache foeniculum* extract and ^d^ sunflower honey is different from the others extracts (*p* < 0.05); Miricetin identified in ^a^ *Vaccinium myrtillus* extract and ^b^ sunflower honey is different from the others extracts (*p* < 0.05); Naringenin identified in ^a^ *Vaccinium myrtillus* extract is different from the others extracts (*p* < 0.05); Kaempferol identified in ^a^ sunflower honey, ^b^ *Laurus nobilis* extract, ^c^ *Vaccinium myrtillus* extract is different from the others extracts (*p* < 0.05); Vanilic acid identified in ^a^ *Evernia prunastri* extract is different from the others extracts or sunflower honey (*p* < 0.05); Vanillin identified in ^a^ *Agastache foeniculum* and ^b^ *Evernia prunastri* extract is different from the others extracts or sunflower honey (*p* < 0.05); Rosmarinic acid identified in ^a^ *Origanum vulgare* and ^b^ *Evernia prunastri* extract is different from the others extracts or sunflower honey (*p* < 0.05); Crisin identified in ^a^ *Evernia prunastri* extract is different from the others extracts or sunflower honey (*p* < 0.05); o-Cumaric acid identified in ^a^ *Evernia prunastri* extract and ^b^ sunflower honey is different from the others extracts (*p* < 0.05); Acacetin identified in ^a^ *Agastache foeniculum* and ^b^ *Evernia prunastri* extract is different from the others extracts or sunflower honey (*p* < 0.05); Gallic acid and Caffeic acid identified in ^a^ *Agastache foeniculum* extract and ^b^ sunflower honey are different from the others extracts (*p* < 0.05); *p*-OH Cinnamic acid identified in ^a^ *Agastache foeniculum* extract is different from the others extracts or sunflower honey (*p* < 0.05); Apigenin identified in ^a^ *Origanum vulgare* extract, ^b^ *Agastache foeniculum* extract and ^c^ sunflower honey is different from the others extracts (*p* < 0.05); Rutin identified in ^a^ *Laurus nobilis* extract, *^b^ Humulus lupulus* extract, ^c^ *Artemisia absinthium* extract and ^d^ sunflower honey is different from the others extracts (*p* < 0.05); Epicatechina identified in *^a^ Humulus lupulus* extract and ^b^ *Laurus nobilis* extract is different from the others extracts or sunflower honey (*p* < 0.05); Vitexin 2-O-ramnoside, Sinapic acid and Resveratrol identified in ^a^ *Origanum vulgare* extract are different from the others extracts or sunflower honey (*p* < 0.05); (Results were analysed using the SPSS programme) ** n.d. –not detectable.

**Table 3:** Antioxidant activity of extracts from *Agastache foeniculum*, *Artemisia absinthium*, *Evernia prunastri*, *Humulus lupulus*, *Laurus nobilis*, *Origanum vulgare*, *Vaccinium myrtillus* and *sunflower honey*. Results are given as mean values of three independent replicates ± standard deviation.

Sample	Radical Scavenging Activity (% Inhibition)	Total Antioxidant Power, CUPRAC Value (µmol TE/g DW)
*Agastache foeniculum*	78.80 ± 1.09 ^b^*	17.96 ± 0.36 ^d^*
*Artemisia absinthium*	97.50 ± 1.05 ^a^*	20.97 ± 0.51 ^d^*
*Evernia prunastri*	76.93 ± 0.86 ^b^*	18.02 ± 0.24 ^d^*
*Humulus lupulus*	46.95 ± 0.31 ^b^*	23.85 ± 0.45 ^c,d^*
*Laurus nobilis*	50.63 ± 0.82 ^d^*	39.16 ± 0.82 ^b^*
*Origanum vulgare*	76.92 ± 0.52 ^d^*	165.59 ± 1.08 ^a^*
*Vaccinium myrtillus*	61.31 ± 0.71 ^c^*	30.99 ± 0.21 ^b,c^*
Sunflower honey	46.99 ± 1.11 ^d^*	22.10 ± 0.40 ^c,d^*

* Different letters within a column denote significant differences (*p* < 0.05). For the CUPRAC value: ^a^ Artemisia absinthium and Origanum vulgare different from the others’ samples (*p* < 0.05); ^b^ *Agastache foeniculum, Evernia prunastri,* *Humulus lupulus, Laurus nobilis* and *Vaccinium myrtillus* different from the others’ samples (*p* < 0.05); ^c^ *Vaccinium myrtillus*, *Humulus lupulus* and sunflower honey were different from the others’ samples (*p* < 0.05); (Results were analysed using the SPSS programme); ^d^ *Laurus nobilis, Origanum vulgare* Sunflower honey, *Agastache foeniculum, Artemisia absinthium, Evernia prunastri* and *Humulus lupulus* different from the others’ samples (*p* < 0.05); TE: Trolox equivalents, DW: dry weight.

**Table 4:** Antimicrobial activity of plant extracts, recipes of medicinal products, negative (80% ethanol–solvent control) and positive (oxytetracycline) controls. Values are given in mm (zone of inhibition), as mean ± SD of triplicate measurements. Different superscript letters show significant differences, as explained below.

Plant Extracts and Recipes	*Paenibacillus* *larvae*	*Paenibacillus* *alvei*	*Brevibacillus* *laterosporus*	*Enterococcus* *faecalis*	*Ascosphaera* *apis*
*Agastache foeniculum*	24.67 ± 1.15 ^a^	18.33 ± 2.88	20.67 ± 1.15	9.33 ± 2.31	9.33 ± 1.15
*Artemisia absinthium*	24.67 ± 4.62	21.33 ± 1.20	21.33 ± 1.15	17.33 ± 2.31	12.67 ± 0.58
*Evernia prunastri*	22.67 ± 2.31	21.33 ± 1.20	18.00 ± 2.00	12.00 ± 2.00	10.67 ± 1.15
*Humulus lupulus*	22.67 ± 4.62	28.67 ± 2.31	22.00 ± 0.00	11.33 ± 2.31	11.00 ± 0.00
*Laurus nobilis*	22.00 ± 0.00	17.33 ± 2.31	10.00 ± 0.00	13.33 ± 1.15	12.00 ± 2.65
*Origanum vulgare*	21.33 ± 2.31	26.00 ± 2.00	20.67 ± 1.15	14.00 ± 0.00	10.67 ± 1.15
*Vaccinium myrtillus*	20.00 ± 0.00	22.67 ± 1.20	13.33 ± 5.77	14.67 ± 2.31	10.00 ± 0.00
Recipe 1	16.67 ± 4.62	26.00 ± 0.00	20.67 ± 1.15	19.33 ± 1.15	10.67 ± 0.58
Recipe 2	21.33 ± 2.31	28.67 ± 1.20 ^b^	24.00 ± 0.00 ^c,d^	14.00 ± 0.00	11.33 ± 2.31
Recipe 3	20.00 ± 0.00	22.67 ± 2.31	22.67 ± 2.31	17.33 ± 2.31	12.67 ± 1.15
Positive control	20.00 ± 0.00	29.33 ± 4.20	20.00 ± 0.00	20.67 ± 1.15	19.33 ± 1.15 ^e^
Negative control	10.00 ± 2.83	11.33 ± 1.20	10.33 ± 1.53	8.50 ± 2.12	8.00 ± 0.00

^a^ *Agastache foeniculum* different from negative control (*p* = 0.037), ^b^ Recipe 2 different from negative control (*p* = 0.049), ^c^ Recipe 2 different from *Laurus nobilis* (*p* = 0.04), ^d^ Recipe 2 different from negative control (*p* = 0.04), ^e^ positive control different from negative control (*p* = 0.028). (Results were analysed using Kruskal–Wallis ANOVA and Bonferroni adjusted Dunn’s post hoc tests).

## Data Availability

The data presented in this study are available in Supplementary Materials.

## Supplementary Materials

The following are available online at https://www.mdpi.com/article/10.3390/antibiotics10080960/s1, Table S1: Plants studied for their biologically active compounds, Table S2: Recipes of medicinal products (final volume 100 mL), Table S3: Microscopic analysis of *N. ceranae-*infected honey bees, Figure S1: Agarose gel electrophoresis of *N. ceranae*-specific PCR products, using spores extracted from “R2” lot samples, Figure S2: Agarose gel electrophoresis of *N. ceranae*-specific PCR products, using spores extracted from “honey” lot samples.
