# Supplementary material for: Biologically Active Extracts from Different Medicinal Plants Tested as Potential Additives against Bee Pathogens
Source: Antibiotics (Basel). 2021 Aug 10;10(8):960. doi: 10.3390/antibiotics10080960 (PMC8388991; doi:10.3390/antibiotics10080960)
Supplement: Supplementary file 1 [file antibiotics-10-00960-s001.zip › antibiotics-1278763-supplementary.pdf]

Supporting Information

Table S1. Plants studied for their biologically active compounds.

Species	Popular name	Part of the plant used	Harvesting month
Agastache foeniculum	anise hyssop	stem, leaves and inflorescences	August
Artemisia absinthium	absinthium	leaves	July
Evernia prunastri	oakmoss	lichen	May
Humulus lupulus var. Brewers Gold	commercial hop	cones	August
Laurus nobilis	bay laurel	leaves	June
Oregano vulgare	oregano	inflorescences	September
Vaccinium myrtillus	blueberry	fruits	July

Table S2. Recipes of medicinal products (final volume 100 mL).

Recipe	Active ingredients (ml)
R1	blueberry extract 50, absinthium extract 10, oakmoss extract 20, oregano extract 10, *Brewers Gold* hops extract 10
R2	blueberry extract 20, absinthium extract 40, oakmoss extract 10, oregano extract 10, *Brewers Gold* hops extract 10, bay laurel extract 5, anise hyssop extract 5
R3	blueberry extract 25, absinthium extract 30, oakmoss extract 15, oregano extract 10, *Brewers Gold* hops extract 15, bay laurel extract 2.5, anise hyssop extract 2.5

Table S3. Microscopic analysis of *N. ceranae* infected honey bees.

Treatment	Cage	*N. ceranae* spores/bee samples (died at 7 days)	*N. ceranae* spores/bee samples (died at 14 days)	*N. ceranae* spores/bee samples (died at 21 days)	*N. ceranae* spores/bee samples (survived at 21 days)
**R1**	**C8**	623,478	356,720	220,965	4,830
**R2**	**C4**	564,375	291,230	200,034	2,391
**R2**	**C7**	513,472	269,012	219,067	2,124
**R3**	**C2**	434,789	325,678	200,123	2,444
**R3**	**C3**	473,126	289,124	210,089	2,731
**Honey**	**C1**	405,625	367,892	205,678	2,895
**Protofil**	**C5**	541,295	270,120	210,890	2,979
**untreated**	**C6**	405,135	589,120	580,101	124,400

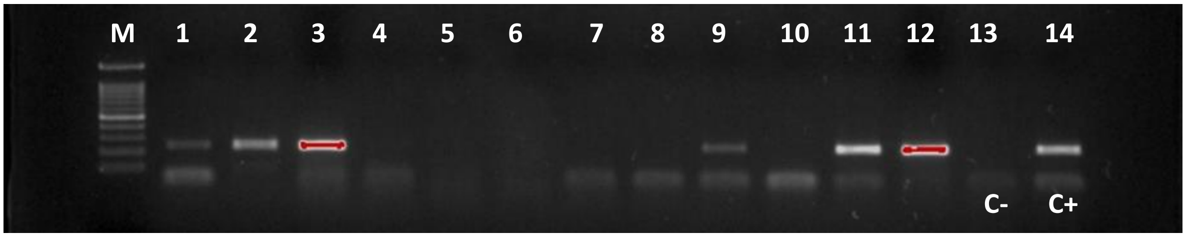

Figure S1: Agarose gel electrophoresis of *N. ceranae*-specific PCR products, using spores extracted from the "R2" lot samples**. (**M - 100bp DNA ladder (100-1500 bp, GeneDireX, Taoyuan, Taiwan); 250 bp - 16S rRNA fragment of *N.ceranae* DNA amplification within the "R2" lot samples: lanes 1-3, 9, 11, 12 positive samples; C- negative control (uninfected bee DNA); C+ positive control (untreated infected bee DNA))

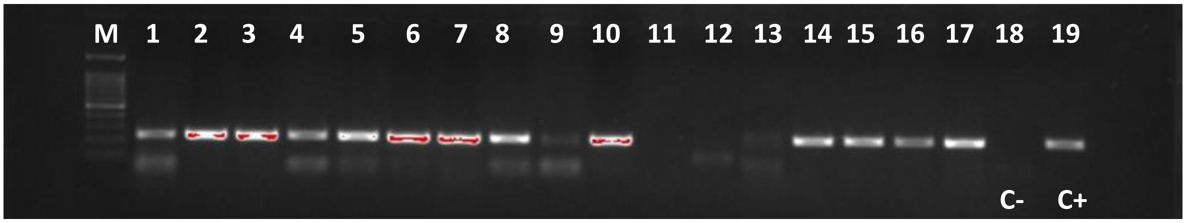

Figure S2: Agarose gel electrophoresis of *N. ceranae*-specific PCR products, using spores extracted from the "honey" lot samples. (M - 100bp DNA ladder (100-1500 bp, GeneDireX, Taoyuan, Taiwan); 250 bp - 16S rRNA fragment of *N. ceranae* DNA amplification within the "honey" lot samples: lanes 1-10,13-17 positive samples (although lanes 9, 13 represent a week amplification, the presence of spores was confirmed by microscopy); C- negative control (uninfected bee DNA), C+ positive control (untreated infected bee DNA))

1. *Total phenolic content determination*

Reagents used: Folin-Ciocalteu reagent (FC) 2M, diluted 1:10 and 7.5% sodium carbonate solution to neutralize and alcalize the reaction medium. The preparation of the calibration curve is done by concentration standard solution: 0.2-0.4-0.6-0.8-1.2 μM/mL gallic acid. The initial yellow solution of FC reagent becomes slightly greenish with the increase in the concentration of gallic acid. After about 10 min, the time necessary to complete the reduction reaction, we added 2 mL of solution 7.5% of sodium carbonate, for neutralizing and alcalizing the reaction medium and the formation of reduced polyphosphomolybdates, colored in blue. The samples analysed (25 μL each) were dissolved in methanol and further diluted in order to be able to make readings on the standard curve made with gallic acid (R = 0.997). The extracts were oxidized by Folin Ciocalteu reagent (120μL) and the neutralization was done with Na_2CO_3 (340 μL) after 5 min. After about 2 h we read the absorbance at 750 nm. The concentration in polyphenols is expressed as gallic acid equivalents.

2. *Total flavonoid content determination*

The extracts were diluted to a final volume of 5 mL with distilled water. After we added 300 μL NaNO2 5%, the samples were left 5 min at rest. Then, 300 μL $AlCl_3$ 10% was added and after 6 min 2ml NaOH 1N was added. The solution was mixed well and absorbance was measured against a control (water) at 510 nm.

3. *Antioxidant activity determination by DPPH method*

The method is based on the decoloration of the stable radical DPPH (2,2'diphenyl-picril-hydrazil), strong dye in red-purple and having the maximum abbreviation between 515-525 nm by anti-radical substances (antioxidant). DPPH solution (80 μM) was prepared fresh in 95% methanol. Protocol: 250 μL solution of DPPH was incubated with 35 μL sample at 30 min and after that we were reading the absorbance at 515 nm.

4. *Antioxidant activity determination by CUPRAC method*

Protocol: 1 mL of concentration 1.0 x 10^{-2} M $CuCI_2 \cdot H_20$, 1 mL of 7,5 x 10^{-3} M Nc and 2 mL of urea buffer pH 7,0. (1.0 $^{-x}$) mL of standard pH 8 buffer solution and 1 mL of sample or standard sample solution were added to this mixture. The final mixture was left at rest at room temperature for 30 min, and the absorbance was read at 450 nm.
